# Ni-rich mineral nepouite explains the exceptional green color of speleothems

**DOI:** 10.1038/s41598-023-41977-7

**Published:** 2023-09-12

**Authors:** Martin Vlieghe, Gaëtan Rochez, Stéphane Pire-Stevenne, Jean-Yves Storme, Augustin Dekoninck, Yves Vanbrabant, Olivier Namur, Yishen Zhang, Alicia Van Ham-Meert, Jean-Pierre Donnadieu, Michel Berbigé, Jean-Luc Hasbroucq, Johan Yans

**Affiliations:** 1 Institute of Life, Earth and Environment, University of Namur, Namur, Belgium; 2 Groupe de Recherches et Photographie en Spéléologie, Namur, Belgium; 3 G-Time Laboratory, Geosciences, Environment and Society Department, Université Libre de Bruxelles, Brussels, Belgium; 4 Geological Survey of Belgium, Royal Belgian Institute of Natural Sciences, Brussels, Belgium; 5 Department of Earth and Environmental Sciences, Katholieke Universiteit Leuven, Leuven, Belgium; 6 Association Mont Marcou, Saint-Geniès-de-Varensal, France

**Keywords:** Geochemistry, Mineralogy, Environmental chemistry

## Abstract

Speleothems are secondary mineral structures typically found in karstic caves and usually composed of calcite or aragonite. Despite being naturally white, some might exhibit unusual colors, such as blue, black, red, yellow or green. The causes of these exceptional colorations are poorly understood, especially for green speleothems, which are barely reported. Here we describe the occurrence of the green Ni-bearing serpentine nepouite in green aragonite and calcite speleothems, in the Aven du Marcou (Hérault, France). Nepouite is mainly found as flat lamellar crystals in the outer rim of green speleothems and crystallized alongside radially grown aragonite crystals. This supports nepouite beginning to crystallize recently, due to a change in the chemical composition of the water. Nepouite also exhibits extensive substitution between Ni, Mg and Zn. The various elements responsible for nepouite precipitation are thought to come from the weathering of pyrite crystals in the overlying rocks, which is consistent with the pH conditions of the cave and the Al-free composition of nepouite. This study explains the crystallization mechanisms and stability conditions of silicate minerals in colored caves.

## Introduction

Aragonite and calcite speleothems, which comprise stalactites and stalagmites, are secondary mineral structures that commonly precipitate from groundwater drip in karstic caves. Despite being naturally white, some exhibit fascinating colors, from jet black to vivid green. Several factors may explain these uncommon colorations, such as the presence of humic and fulvic acid^1,2^, substitution of metals for Ca in carbonates (e.g. Cu, Cr, Mn, Ni)^3–5^, variations in the texture of the speleothems, leading to light wave interferences^6^, trapped organic matter^7^ or the presence of other colorful mineral phases^3^.

Speleothems colored by these factors are usually red, yellow, brown, black, blue, or green. Despite being visually stunning, green speleothems are poorly understood, partly because studying colored caves often requires advanced speleological skills, but mostly because this green coloration is very unusual. The Aven du Marcou constitutes a major occurrence of green speleothems. To our knowledge, the only published occurrence of green speleothems is in Timpanogos Cave National Monument, Utah, and their origin and formation process are not clearly stated^3^. This scarcity highlights the significant lack of understanding of these natural structures, making them a valid case for further study.

Here we determine the causes of green coloration in aragonite and calcite speleothems in the Aven du Marcou (Hérault, France), and we propose an explanation of their origin. Specifically, we explain the timing of crystallization in the green structures, the physico-chemical conditions determining the transport and precipitation of chromophore elements, and the primary source of these elements.

The Aven du Marcou is located in Southern France, north of the Montagne Noire Massif, which is part of the Variscan Belt^8^. It extends into the Lower Cambrian dolomitic Marcou Formation, in the sedimentary nappe called Lacaune Mountains^9^. The cave is near a large thrust fault, responsible for the dense fracturing of the neighboring rocks. It is partly overlapped by the Lower Cambrian pyrite-bearing mudstone and sandstone Marcory Formation^10^ (Supplementary Fig. 1 online).

## Results

**Figure 1:**
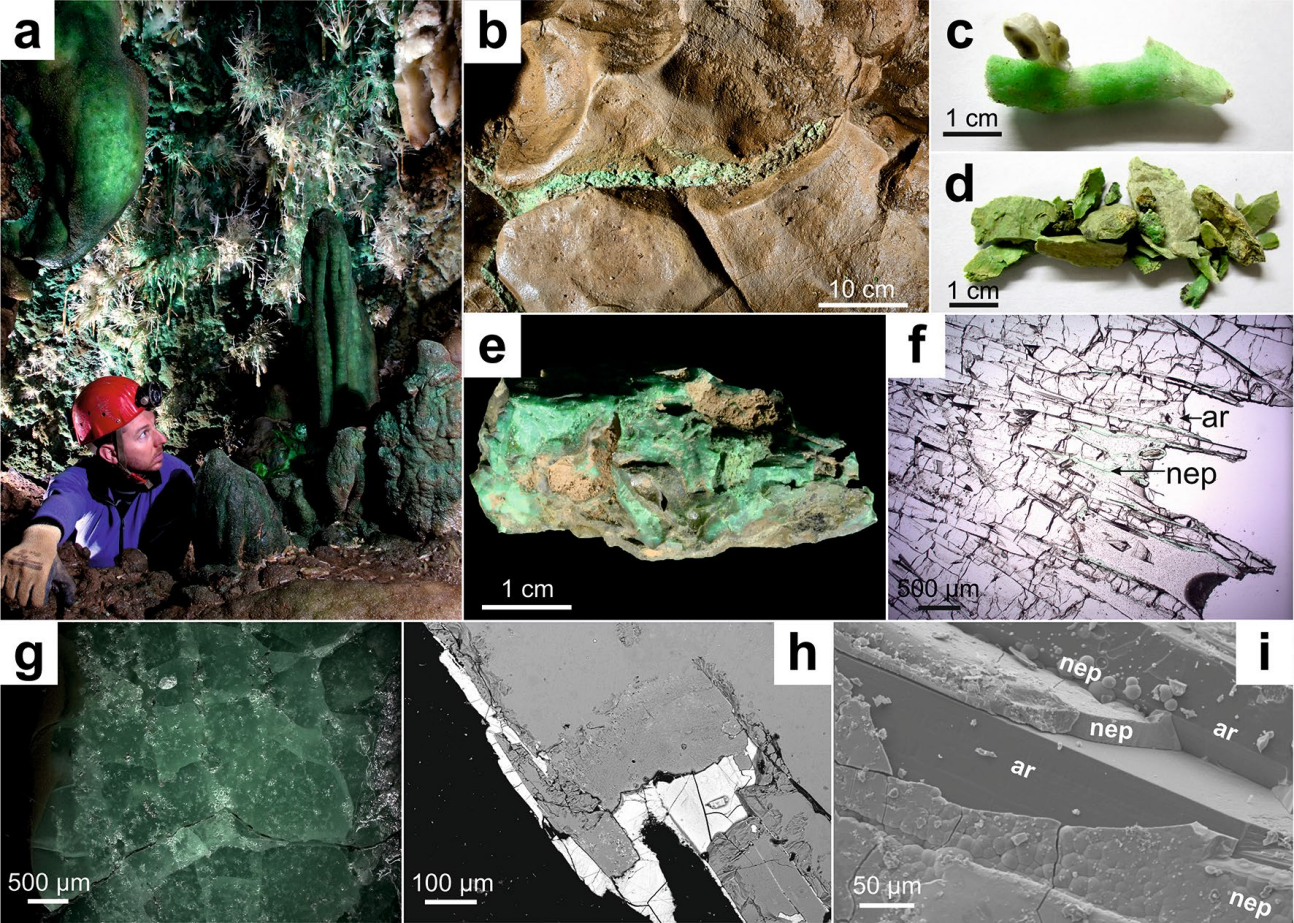
Macroscopical and microscopical observations. (**a**) Main room containing green speleothems in the Aven du Marcou, France. (**b**) Green clay vein within host dolostone. (**c**) Green speleothem sample. (**d**) Green clay sample. (**e**) Green clay sample with dolostone. (**f**) Transmitted light view of aragonitic green speleothem. *ar* aragonite, *nep* nepouite. (**g**) Reflected light view of a green clay sample. (**h**) Backscattered electron view of nepouite at the edge of aragonite in a speleothem sample. (**i**) Secondary electron view of nepouite coating aragonite crystals in a speleothem sample.

Green speleothems in the Aven du Marcou are located within a 5 m wide chamber, at a depth of 70 m from the cave’s entrance, and in a smaller chamber just above it. Green and white stalactites and stalagmites (Fig. 1a) as well as clay-filled fractures (Fig. 1b) are found in this chamber.

For this study, green (Fig. 1c) and white stalactite samples were collected, along with several green clay fragments (Fig. 1d,e) and dolomitic bedrock fragments. All samples were picked up from the cave’s floor, to preserve the exceptional speleothems. To investigate the origin of chromophore elements, several samples were also taken outside of the cave, from the overlying Marcory Formation (Supplementary Fig. 2 online).

### Microscopical observations

Optical microscopy observations on thin or polished rock sections show the presence of a green mineral phase in green speleothems and clay samples, whereas this phase is completely absent in white speleothem samples. These minerals consist of 5–10 μm wide lamellae (Fig. 1f) or botryoidal aggregates in green speleothems, or as coarsely crystallized masses in clay samples (Fig. 1g).

In aragonite speleothem samples (Fig. 1f), the lamellae are disposed radially, along with the aragonite crystals, and always within a few millimeters of the outer rim of the stalactite. However, in the calcite speleothems neither the calcite crystals nor the green lamellae seem to exhibit any preferential direction, although the latter are still found near the outer rim of the samples.

### Speleothem mineralogy

**Figure 2:**
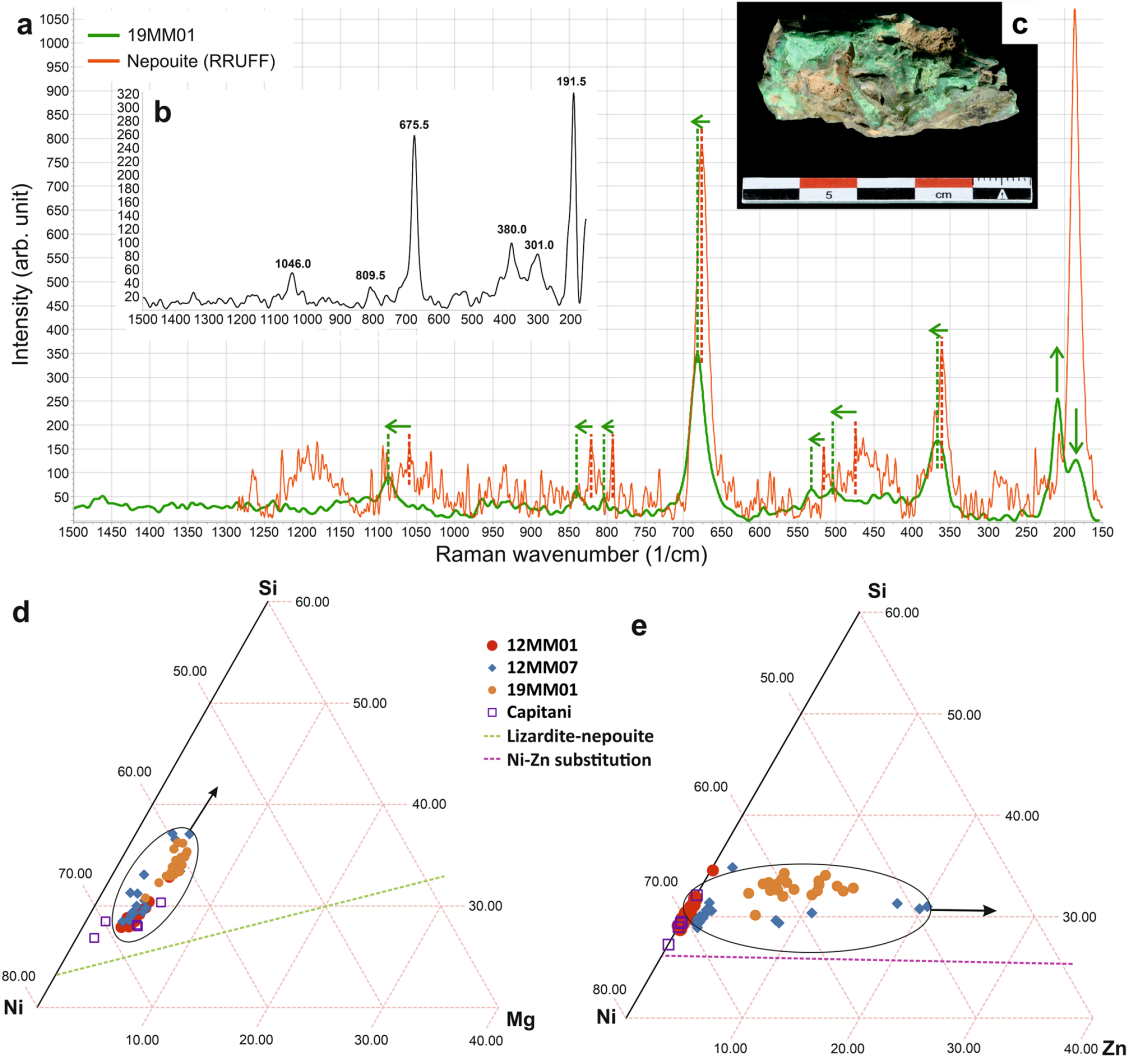
Mineralogical and geochemical results. (**a**) Comparison of the Raman spectrum of sample 19MM01 and the RRUFF project nepouite reference spectrum^13^. Green arrows emphasize differences between the RRUFF spectrum and sample 19MM01. (**b**) Spectrum obtained on a nepouite crystal from a sample of the Royal Belgian Institute of Natural Sciences collection. Raman shift values of the major peaks are displayed. (**c**) Sample 19MM01 from which the experimental spectrum was obtained. (**d**) Mg–Si–Ni and (**e**) Zn–Si–Ni relative grades ternary diagrams obtained by EPMA and compared with results from literature^12^. Ellipses and arrows emphasize the general tendency observed for samples 12MM07 and 19MM01. Lizardite-nepouite solid solution^15^ (**d**) and stoechiometric Ni–Zn substitution in nepouite (**e**) are also displayed.

The X-ray diffraction (XRD) spectrum of sample 19MM01 (Supplementary Fig. 3 online), which contains a 5 cm green vein, shows a clear match with reference nepouite and/or pecoraite, whose XRD spectra are very similar^11,12^. Raman microanalyses confirm the presence of nepouite in the speleothem samples (Fig. 2a–c).

Comparisons with reference spectra showed a close match, but each peak of our samples exhibits a slight shift (5–30 cm^−1^) towards higher Raman wavenumbers, and the peaks located c. 180 and 210 cm^−1^ present a strong intensity inversion (Fig. 2a). The variations may be attributed to various factors, such as a difference in the incident laser wavelength, crystal defects, different techniques during sample preparation, chemical substitutions and others. The sample’s Raman spectrum is also much closer to reference nepouite spectra^13^ than to reference pecoraite spectra^14^.

### Speleothem geochemistry

Inductively coupled plasma mass spectrometry (ICP-MS) analyses on bulk green speleothem samples confirm that these speleothems are enriched in chalcophile elements: 3420–27,300 ppm Ni as well as Mg (300–2600 ppm), As (132–792 ppm), Sb (0.3–2.7 ppm) and Pb (5–12 ppm). These results are higher than the white speleothem grades (80 ppm Ni, 60 ppm Mg, 187 ppm As, 0.3 ppm Sb and < 5 ppm Pb). We therefore conclude that all these elements are linked to nepouite rather than pure aragonite or calcite.

Backscattered electron views obtained by scanning electron microscopy (SEM) show an important compositional contrast between aragonite and nepouite (Fig. 1h), as the latter contains heavier chemical elements than the former (mostly Ni). The phases shown on the secondary electron view (Fig. 1i) exhibit a flat and lamellar morphology, typically observed in nepouite and different from pecoraite morphology, which is cylindrical^11,12^. Electron probe micro analysis (EPMA) performed on several nepouite phases from three separate samples allowed us to determine general structural formulae for nepouite from each sample (Table 1).

**Table 1:** Nepouite structural formulae based on EPMA measurements of 3 nepouite-bearing samples.

Sample	Formula
12MM01	(Ni_2.20–2.43_Mg_0.29–0.41_Zn_0.00–0.01_)Si_2.06–2.16_O_5_(OH)_4_
12MM07	(Ni_1.63–2.37_Mg_0.20–0.44_Zn_0.04–0.61_)Si_2.06–2.25_O_5_(OH)_4_
19MM01	(Ni_1.77–2.14_Mg_0.29–0.43_Zn_0.13–0.38_)Si_2.11–2.26_O_5_(OH)_4_

12MM01 and 12MM07 are green speleothems and 19MM01 is a green clay vein.

All formulae were calculated considering 14 negative charges. These formulae agree solidly with the theoretical formula for nepouite [(Ni,Mg)_3_Si_2_O_5_(OH)_4_)]^15^. However, the Zn grade is too high in samples 12MM07 and 19MM01 to be considered to be traces (up to 11.5 wt% Zn, see Supplementary Data). Such a high grade of Zn in nepouite constitutes a newly discovered substitution range, as Zn is always reported as traces in other nepouite deposits (about 200 ppm (Campello Monti)^12^, 28–248 ppm (Sulawesi Island)^16^). Si, Ni, Mg and Zn proportions are displayed in Fig. 2d,e. Measurements from sample 12MM01 lie close to the theoretical lizardite-nepouite isomorphous series (Mg substituting for Ni)^15^. Those from samples 12MM07 and 19MM01 diverge strongly from it, with a very clear Zn substitution for Ni at a constant Mg grade.

To unravel the origin of metals in nepouite, a geological survey was carried out at Mont Marcou. Specifically, water circulation from the surface was studied, and the bedrock was characterized and sampled. Water sources were surveyed (Supplementary Fig. 2 online) and chemically analyzed (Supplementary Table 1 online). The one located closest to the cave chambers where nepouite is found, labeled MMA 3, contains a Ni concentration of 320 ppm, compared to less than 10 ppm in every other source sampled. The search for the origin of this high Ni grade led to the discovery of pyrite-rich zones within the Marcory formation. X-ray fluorescence analyses on those pyrite-bearing sandstone samples showed significative grades of Ni (up to 453 ± 134 ppm), Zn (up to 87 ± 9 ppm) and As (up to 204 ± 16 ppm).

## Discussion

Nepouite is the mineral phase at the origin of the green color of speleothems in the Aven du Marcou. Such Ni-bearing phyllosilicates are most commonly found in serpentinite hydrothermal and/or supergene alteration profiles of ophiolitic rocks (e.g. ^17–20^). This section aims to provide an explanation of the processes that could explain its formation in the Aven du Marcou.

Nepouite is found in green clay-filled fractures (Fig. 1b), suggesting the migration of coloring elements in fluids drained through the karst and fracture system. In speleothems, nepouite is always observed near the stalactites’ outer rim, showing that it crystallizes near the most recent part of the speleothems’ formation process, as stalactites usually crystallize radially from a central canal towards the outer rim^21^. This indicates that seeping water underwent an important change in its chemical composition. The nature and causes of this change are still unknown and require further investigation. Si and several chalcophile elements were transported, resulting in nepouite crystallization. As radial aragonite in speleothems is often intercalated with nepouite crystals (Fig. 1f), these two phases probably crystallized jointly. However, calcite crystals in speleothems do not show any preferential direction. Calcite crystallized around nepouite crystals, evidence of posterior crystallization of the former. The absence of preferential direction is linked to secondary calcite that forms by dissolution and recrystallisation of primary aragonite crystals^22^.

Nepouite from the Aven du Marcou is quite different from other nepouite described in the literature, particularly considering the high Zn grade in some of the samples. Furthermore, the trend in Fig. 2e is very similar to a stoichiometric substitution between Ni and Zn, up to a Ni–Zn ratio of about 80–20, respectively. Nepouite is often reported to exhibit Ni–Mg substitution in the tetrahedral sites, constituting the lizardite-nepouite series (e.g. ^15,16,20^), and Ni–Zn substitution is theoretically possible, as the ionic radii of Ni and Zn are quite close to each other in tetrahedral sites (respectively 0.565 Å and 0.580 Å)^23^ and their chemistry is very similar, both being chalcophile elements with a similar atomic mass. Although few minerals exhibit extensive Ni–Zn substitution (e.g. ankinovichite^24^), this substitution is still possible in nature. Substitution in silica tetrahedral sites can also explain the slight difference in Raman spectra compared to end-member nepouite, as previously observed for olivines^25,26^, garnets^27,28^ or feldspars^29,30^.

The origin of chalcophile elements forming nepouite may be discussed, in the light of regional geology. First of all, even though the Aven du Marcou exhibits traces of fossil hydrothermal mineralizations (e.g. quartz veins), these formed prior to karstification, as quartz veins cut across whole chambers in now-dissolved dolostone host rock.

The Marcou Formation is not known to host extensive chalcophile element ores^9^, and chemical analyses performed on the host dolostone within the Aven du Marcou show that it does not contain high amounts of Si (max 2.34 wt%), Ni (< 20 ppm) or any other chalcophile element. However, as stated before, the Aven du Marcou is located near a major thrust fault and is partly overlapped by the mudstone and sandstone Marcory Formation^10^.

**Figure 3:**
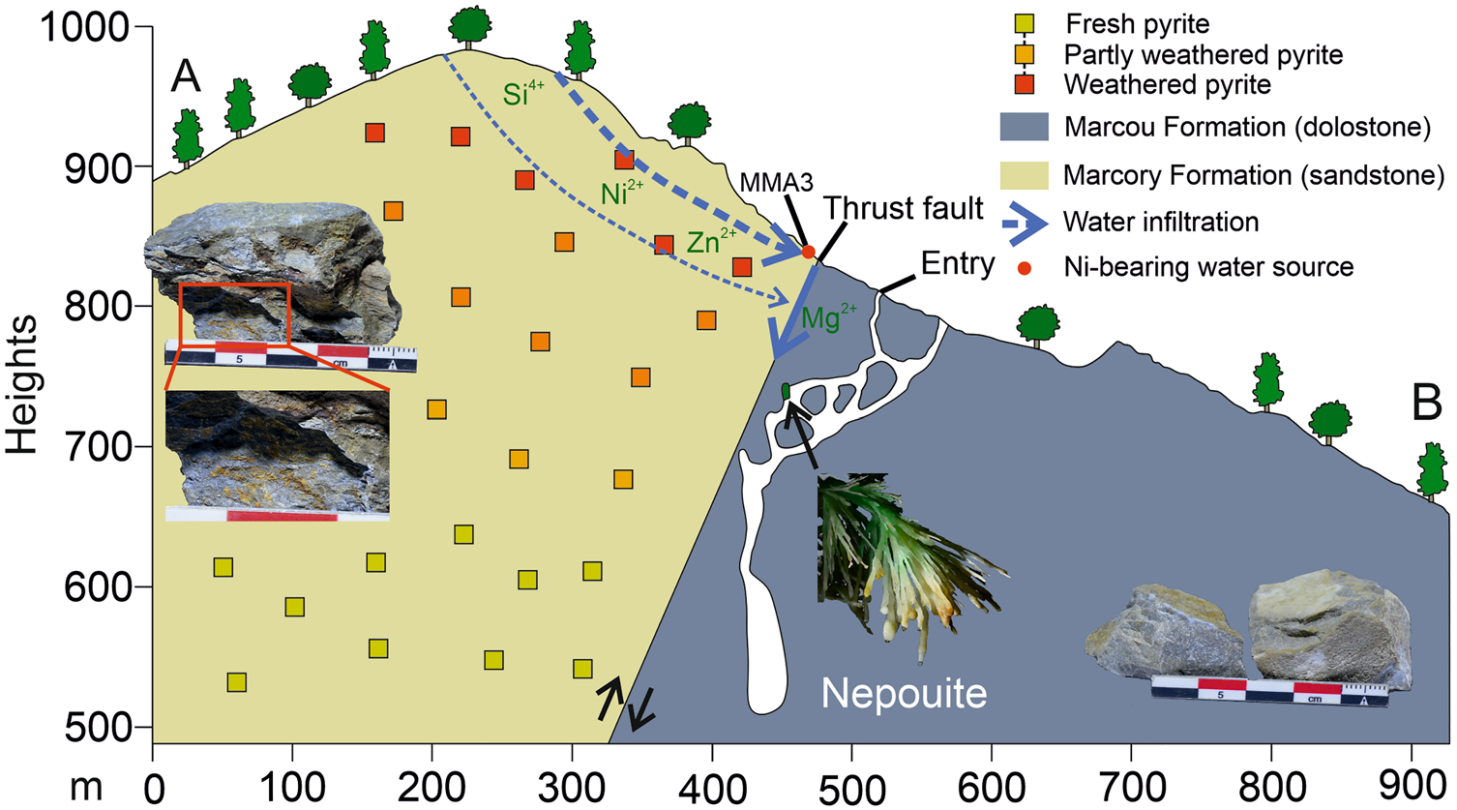
(**A**,**B**) Cross-section (see Supplementary Fig. 2 online for localization) of the Aven du Marcou illustrating the weathering, oxidation and leaching processes of the pyrites, and associated elements migration to the cave.

Field observations show that these rocks contain significant amounts of pyrite crystals, showing extensive traces of weathering (Fig. 3). Bulk ICP-MS analyses emphasize significant grades in various chalcophile elements in these rocks (up to 70 ppm Ni, 40 ppm Cu, 80 ppm Zn, 11 ppm As), as classically found in other weathered pyrite sites (e.g. Waterloo, Agincourt and Rosebery deposits, eastern Australia^31^).

Moreover, chemical analyses of water from the various sources and resurgences of Mont Marcou (Fig. 3, Supplementary Fig. 2 online) show one source having a high Ni concentration (about 320 ppm, Extended Data Table 1). This source almost exactly overlies the cave chambers where nepouite is observed. Therefore, we propose that the main source of the Si, Ni, Mg, Zn and other chalcophile elements in nepouite from Mont Marcou is related to the weathering of the overlying pyrites from the Marcory Formation. These elements were transported to the cave by meteoric water through densely fractured host rock, favored by the presence of a large thrust fault (Fig. 3)^32^. The high compositional variability in the samples described above could be explained by compositional differences between leached pyrites, leading to spatial heterogeneities in the groundwater.

Pyrite dissolution generates fluid acidification by forming sulfuric acid^33–35^. In such acidic and oxidizing conditions, the chalcophile elements contained in pyrite are easily leached out of their host rock. In karstic environments, these fluids are directly neutralized by the cave’s carbonate host rocks^36^. Al solubility is strongly pH-dependent, being high at low pH and becoming low near neutral conditions^37,38^.

During the dissolution of pyrite in the dolomitic bedrock of the Aven du Marcou, Al, which is quite concentrated in the host rock (8.9 wt% Al), is no longer able to migrate through massive dolostones due to neutral conditions, and precipitates rapidly within clay minerals in the fractures. This generates nepouite, an Al-free phyllosilicate, in the cave, and subsequently creates a green color in the speleothems. Furthermore, typical physico-chemical conditions needed to precipitate nepouite rather than its polymorph are consistent with typical cave environments, as nepouite, to crystallize, requires both alkaline conditions and the lowest SiO_2_ activity of all Ni-bearing phyllosilicates^39^. This further consolidates our hypothesis.

As this study describes such a unique occurrence, it might lead to several new studies in the Aven du Marcou and in other colored (e.g. blue, yellow, red) caves. For instance, the mineralization timing of speleothems could be improved by dating the different minerals found in the cave. The refined origin of the Ni-rich fluids responsible for nepouite crystallization could be further confirmed by stable isotope analyses and tracer tests.

This study brings key geological and geochemical constraints to the formation of exceptional green speleothems and is a key step in understanding secondary Ni mineralization in the Aven du Marcou, and others yet to be discovered. This will, in turn, lead to new insights about metallic element transportation in karstic environments.

## Methods

### Optical microscopy

Optical microscopy observations were made at University of Namur on standard polished thin sections (thickness 30 μm, for transmitted light observations) and on samples mounted in epoxy resin and polished (for reflected light observations), using a Zeiss Axiophot petrographical polarizing microscope coupled with a Euromex camera. Both transmitted and reflected light observations were made.

### Scanning electron microscopy

Polished sections were observed at University of Namur using a JEOL-7500F scanning electron microscope coupled with an Ultra Nine 30 JED-2300F energy dispersive X-ray spectrometer (EDS). The samples were coated with ~ 20 nm of carbon using a Quorum Q150 T/ES to ensure good conductivity and avoid charge effects. Acceleration voltage was 20 kV. Observations were made using a backscattered electrons detector. Semi-quantitative EDS analyses were obtained using the standardless method and ZAF correction.

### X-ray diffraction

The samples were grounded with a RETSCH PM 100 planetary ball mill, using opal bowls and marbles to avoid metal contamination. The powdered sample were then sieved using a 125 μm mesh. X-ray diffraction (XRD) analysis was performed at University of Namur using an X-ray panalytical X’Pert Pro diffractometer and a PHILLIPS PW3710 (CuKα radiation) at the PC^2^ platform (UNamur), operating at 40 kV and 30 mA in the 5°–70° 2θ range. CuKβ radiation was filtered out using a Ni filter placed along the diffracted beam path.

### Raman microanalysis

Samples 12MM01, 12MM07 and 19MM01 were analyzed by Raman microanalysis, which were performed using a Bruker Senterra microspectrometer built around an Olympus petrographical microscope (Royal Belgian Institute of Natural Sciences). Incident laser wavelength was 785 nm, laser power was 10 mW, integration time was 20 s and laser aperture 50 × 1000 μm. The determination of Raman spectra was performed by comparison with the RRUFF database. The RRUFF is a free database containing over 20,000 Raman spectra at different excitation wavelengths (514.5, 532, 780 and 785 nm), allowing a quick and robust comparison with experimental data^13^. Experimental spectra were also compared with a spectrum obtained on a nepouite sample from the collection of the Royal Belgian Institute of Natural Sciences, using the same acquisition parameters.

### Inductively coupled plasma mass spectrometry and optical emission spectrometry

Powdered and sieved samples were geochemically analyzed at Activation Laboratories (Ancaster, Canada) by Inductively Coupled Plasma Mass Spectrometry (ICP-MS) and Inductively Coupled Plasma Optical Emission Spectrometry (ICP-OES) to obtain their bulk geochemical composition. Sample preparation was performed by lithium metaborate/tetraborate fusion and digestion of the molten beads in weak nitric acid, as well as some additional analyses (e.g., CO_3_^2−^ content, organic matter) by infrared spectroscopy.

### Electron probe microanalysis

Polished sections 12MM01, 12MM07 and 19MM01 were analyzed by Electron Probe Microanalysis (EPMA) with a JEOL JXA-8530F equipped with a field emission electron gun (FEG), at the Department of Materials Engineering of KU Leuven (Leuven, Belgium). Acceleration voltage was 20 kV and probe current 30 nA. Focus beam was used during the analysis. Peaking counting time is 10 s and half on the background. The following primary standards were used for calibration: Ni metal (Ni), diopside (Si, Ca), albite (Al), skutterudite (As), hematite (Fe), jadeite (Na), olivine (Mg), sphalerite (Zn) and copper metal (Cu).

### X-ray fluorescence

Pyrite-bearing samples were analyzed by X-ray fluorescence analysis, performed using a FONDIS Electronic NITON XL3t 960 spectrometer operating at 50 kV maximal tension and 100 μA maximal current at University of Montpellier, France.

### Water analyses

Water samples were chemically analyzed at Wessling Laboratories (Paris, France) by 3 different methods: (1) metals were quantified by an Agilent ICP-MS according to the NF EN ISO 17294-2 norm. Water samples were mineralized and nebulized before the ICP-MS analysis. (2) Silica was quantified by photometry according to the DIN 38405 D21 method. (3) Sulfate ions were quantified using a Thermo Scientific ion chromatography system according to an intern method.

### Supplementary Information

Supplementary Information 1.

Supplementary Information 2.

## Data Availability

Additional data, comprising a complete list of the analyzed samples and relevant EPMA results, are available in the figshare.com data repository at https://doi.org/10.6084/m9.figshare.23052329.v2. Microscopical images and sample pictures are available from the corresponding authors on request.
