# Supplementary material for: Ni-rich mineral nepouite explains the exceptional green color of speleothems
Source: Sci Rep. 2023 Sep 12;13:15017. doi: 10.1038/s41598-023-41977-7 (PMC10497518; doi:10.1038/s41598-023-41977-7)
Supplement: Supplementary file 1 — Supplementary Information 1. [file 41598_2023_41977_MOESM1_ESM.pdf]

Supplementary information for "Ni-rich mineral nepouite explains the exceptional green color of speleothems"

Martin Vlieghe*, Gaëtan Rochez, Stéphane Pire-Stevenne, Jean-Yves Storme, Augustin Dekoninck, Yves Vanbrabant, Olivier Namur, Yishen Zhang, Alicia Van Ham-Meert, Jean-Pierre Donnadieu, Michel Berbigé, Jean-Luc Hasbroucq, Johan Yans*

*Corresponding authors: martin.vlieghe@unamur.be; johan.yans@unamur.be

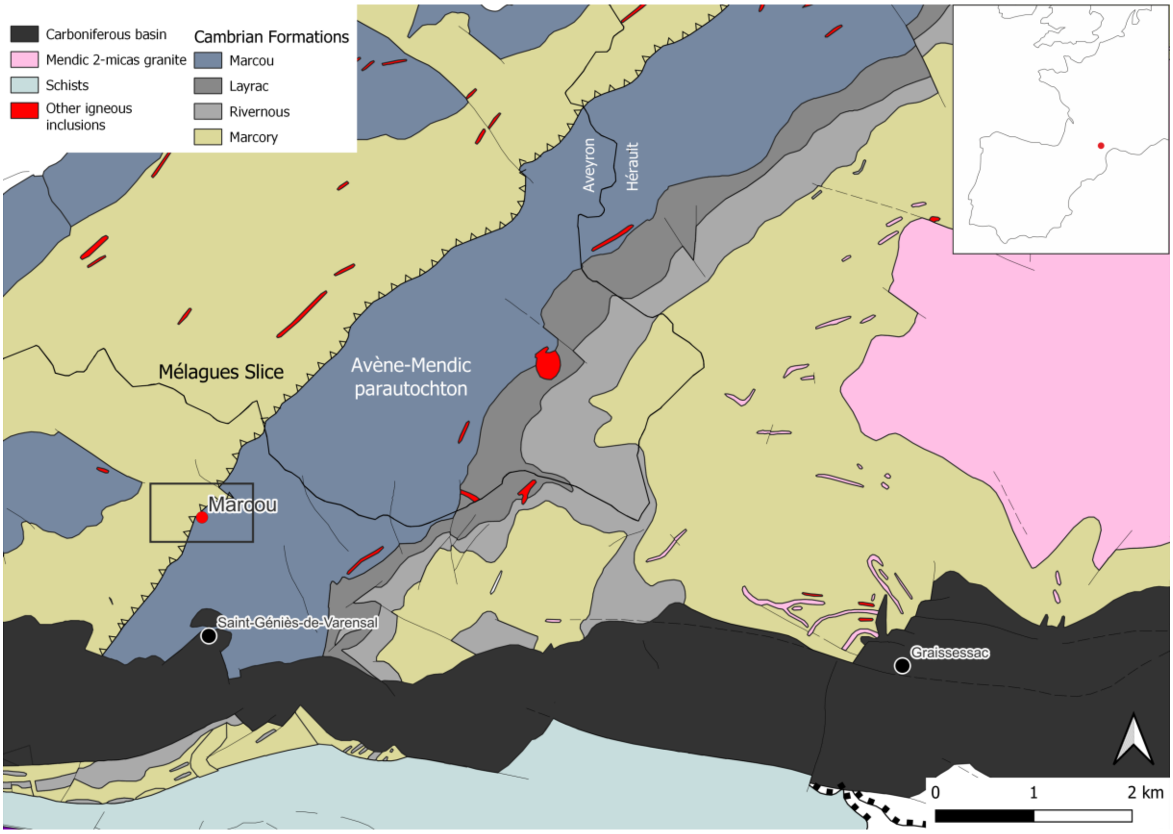

Supplementary Figure 1. Regional geology of the Aven du Marcou. See Supplementary Fig. S2 for a close-up of the cave's surroundings (black rectangle). The map was generated using the QGIS software (version 3.24), see https://www.qgis.org/en/site/.

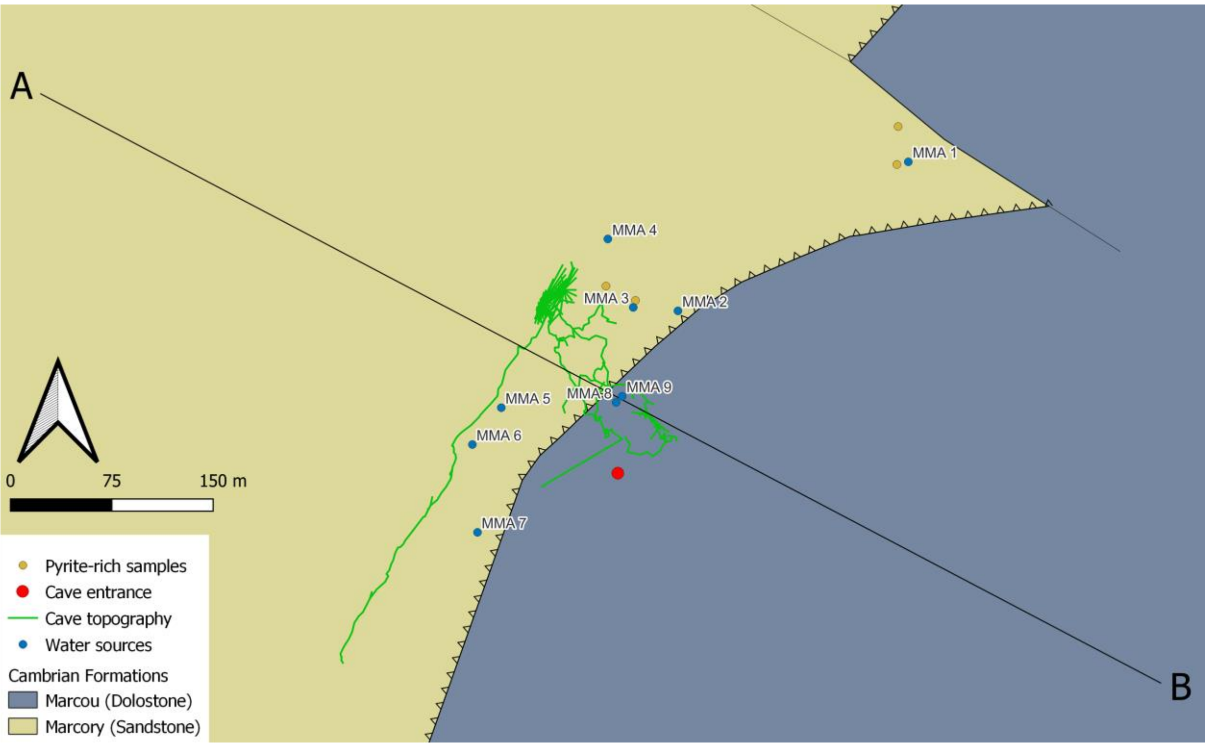

Supplementary Figure 2. Cave topography and localization of the sampled water sources. See Fig. 3 for A-B cross-section. Sampling sites for pyrite-bearing rocks are also shown. The map was generated using the QGIS software (version 3.24), see https://www.qgis.org/en/site/.

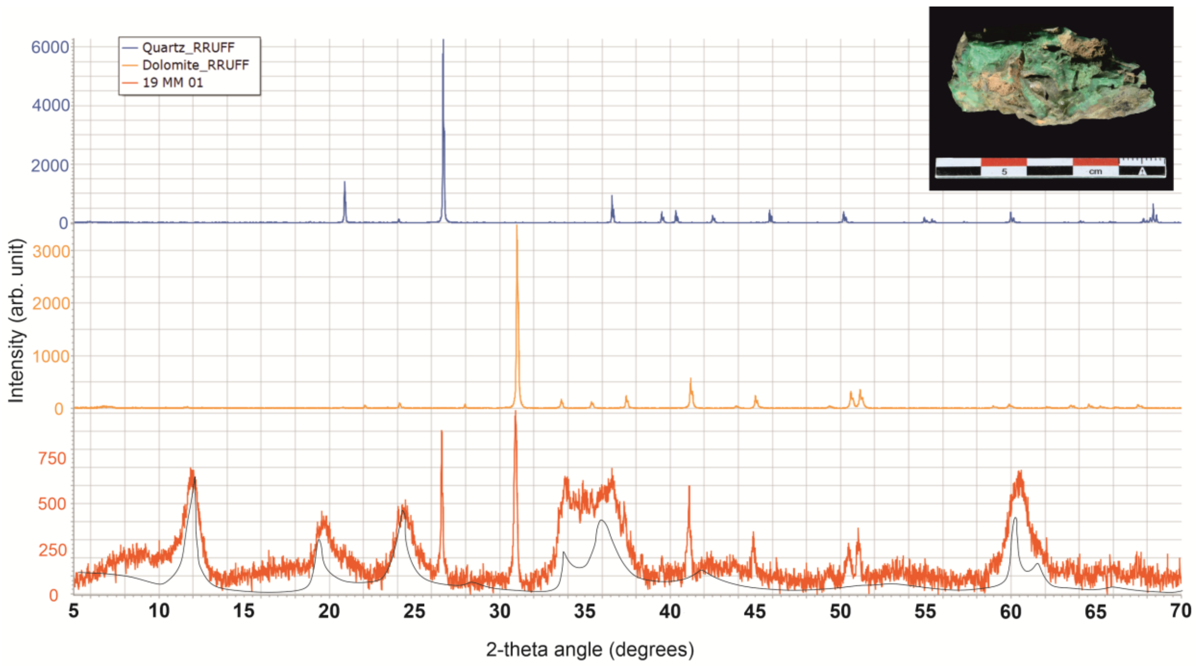

Supplementary Figure 3. Whole rock X-Ray Powder Diffraction spectrum of sample 19MM01 compared to reference quartz and dolomite spectra[13]. The black superimposed spectrum corresponds to a reference nepouite spectrum from the literature[11], and highlights a good match with the data.

Supplementary Table 1. Chemical concentrations in the 9 sampled water sources (see Supplementary Fig. 2).

	**Unit**	MMA 1	MMA 2	MMA 3	MMA 4	MMA 5	MMA 6	MMA 7	MMA 8	MMA 9
SO_4^{2-}	mg/l E/L	12	13	11	5	7	130	9	21	23
Mg	mg/l E/L	3.5	5	2.8	2.8	2.3	2	2.2	4.4	6.2
P	µg/l E/L	<30	<30	<30	<30	<30	<30	<50	<30	<30
Ca	mg/l E/L	18	24	16	16	16	68	20	20	23
V	µg/l E/L	<5.0	<5.0	<5.0	<5.0	<5.0	<5.0	<5.0	<5.0	<5.0
Cr	µg/l E/L	<5.0	<5.0	<5.0	<5.0	<5.0	<5.0	<5.0	<5.0	<5.0
Ni	µg/l E/L	<10	<10	320	<10	<10	<10	<10	<10	<10
Cu	µg/l E/L	<5.0	<5.0	<5.0	<5.0	<5.0	<5.0	<5.0	<5.0	<5.0
Zn	µg/l E/L	<50	<50	<50	<50	<50	<50	<50	<50	<50
As	µg/l E/L	<3.0	<3.0	<3.0	<3.0	<3.0	<3.0	<3.0	<3.0	<3.0
Sr	µg/l E/L	58	110	68	65	75	410	82	71	72
Ba	µg/l E/L	<5.0	<5.0	<5.0	<5.0	7	<5.0	<5.0	<5.0	<5.0
Pb	µg/l E/L	<10	<10	<10	<10	<10	<10	<10	<10	<10
Fe	mg/l E/L	<0.05	<0.05	0.17	<0.05	<0.05	<0.05	<0.05	<0.05	<0.05
